# Prognostic value, immune signature and molecular mechanisms of the APOBEC family members APOBEC1, APOBEC3A, APOBEC3G and APOBEC3H in pancreatic adenocarcinoma

**DOI:** 10.3389/fmolb.2022.1036287

**Published:** 2022-10-20

**Authors:** Yunjie Duan, Yongxing Du, Zongting Gu, Xiaohao Zheng, Chengfeng Wang

**Affiliations:** ^1^ State Key Lab of Molecular Oncology and Department of Pancreatic and Gastric Surgery, National Cancer Center/National Clinical Research Center for Cancer/Cancer Hospital, Chinese Academy of Medical Sciences and Peking Union Medical College, Beijing, China; ^2^ Department of Hepatobiliary and Pancreatic Surgery and Minimally Invasive Surgery, Zhejiang Provincial People’s Hospital, Hangzhou Medical College, Hangzhou, China

**Keywords:** pancreatic adenocarcinoma, APOBEC family, biomarkers, prognosis, immune infiltration

## Abstract

**Background:** Increasing evidence supports that the APOBEC family is associated with development of a variety of cancers. However, the function of APOBEC1/3A/3G/3H in pancreatic adenocarcinoma (PAAD) is still unclear.

**Methods:** Comprehensive bioinformatic analysis using R (version 3.6.3), TISIDB, Metascape etc. were performed to study the clinicopathological characteristics, prognostic value, immune features and functional mechanisms of the APOBEC1/3A/3G/3H in PAAD.

**Results:** APOBEC1/3A/3G/3H showed significantly elevated expression in PAAD than para-cancerous or normal tissues. Their high expression or amplification were significantly correlated with worse clinicopathological characteristics and prognosis in PAAD patients. In addition, the role of APOBEC1/3A/3G/3H in the immune regulation is diverse and complex, the high expression of APOBEC1 may inhibit the infiltration level of many kinds of immunoreactive tumor-infiltrating cells, which may be an important factor leading to immune escape of PAAD cells. Mechanistically, APOBEC1/3A/3G/3H played an activating role in multiple oncogenic pathways, including the EMT, RAS/MAPK and TSC/mTOR pathways. Moreover, we found that the expression level of APOBEC3G was positively correlated with the sensitivity of gemcitabine and doxorubicin.

**Conclusion:** APOBEC1/3A/3G/3H play an oncogenic role in the development of PAAD and might serve as new biomarkers or therapeutic targets.

## Introduction

Pancreatic adenocarcinoma (PAAD) is one of the most invasive and lethal human cancers (Ioannou et al., 2012). It is highly malignant, prone to distant metastasis and has a poor prognosis. The 5-year survival rate of patients is only 7.2% (He et al., 2022). As per global reports in 2018, pancreatic adenocarcinoma remains the twelfth most common cancer in men and the eleventh in women. Cancer-related deaths have pancreatic adenocarcinoma as the seventh leading cause (He et al., 2022). Complete surgical resection is the only cure, but due to the lack of specific pancreatic adenocarcinoma screening methods, less than 20% of patients are diagnosed with local tumors, and most pancreatic adenocarcinoma patients cannot receive surgical treatment (Lin et al., 2019). Mucin-related markers such as CA19-9 are the most widely used tumor markers of pancreatic adenocarcinoma, but their value as screening markers is limited by their low specificity (Ringel and Löhr, 2003). Therefore, it is very important to explore new biomarkers and therapeutic targets to improve the prognosis of patients with pancreatic adenocarcinoma.

The apolipoprotein B mRNA-editing catalytic polypeptide (APOBEC) family has 11 members, including APOBEC1, APOBEC3A, APOBEC 3G and APOBEC 3H (Wang et al., 2008), all of which have a zinc-dependent cytidine deaminase domain (ZDD) (Smith et al., 2012). The expressed product is a highly efficient cytidine deaminase that can convert cytosine to uracil. It can also be used as an inhibitor of retroviral replication and retrotransposon migration through deaminase-dependent and deaminase-independent mechanisms. APOBEC1 can edit apolipoprotein B mRNA to regulate cholesterol metabolism, and its expression products have a carcinogenic effect on abnormal deamination of genomic DNA (Wolfe et al., 2020). In addition, APOBEC1 is also a potential endogenous mutation factor (Niavarani et al., 2018). Changes related to APOBEC1 mutation activity may enhance its carcinogenic potential (Saraconi et al., 2014) and promote the occurrence and development of many cancers, including gastrointestinal tumors and esophageal cancer (Niavarani et al., 2018; Buisson et al., 2019). APOBEC3A is the only APOBEC enzyme that has a great preference for hairpin substrates (Buisson et al., 2019). It is the cause of genetic heterogeneity in tumors and has become the main driver of cancer cell mutagenesis. Its overexpression can lead to DNA damage (Biayna et al., 2021) and inducement of invasive breast cancer (Kim et al., 2020). APOBEC3G is closely related to increased DNA damage and DNA repair disorders. Misregulation of its expression products can lead to somatic mutations in many cancers and affect the stability of the genome (Iizuka et al., 2017). Related studies have found that high expression of APOBEC3G can promote the occurrence and development of a variety of malignant tumors, including hepatocellular carcinoma, multiple myeloma and esophageal squamous cell carcinoma (Wang et al., 2008; Ding et al., 2011; Talluri et al., 2021). The expression of APOBEC3H in head and neck squamous cell carcinoma is upregulated and may regulate the immune response through its demethylation activity, which has been identified as a potential target for immunotherapy of head and neck squamous cell carcinoma (Liu J et al., 2020). In addition, its strong retroviral restriction and hypermutation activity are also the main causes of breast cancer and lung cancer cell mutation (Starrett et al., 2016; Liu Q et al., 2020). However, the role of APOBEC1, APOBEC3A, APOBEC3G and APOBEC3H in pancreatic adenocarcinoma is not clear, and related studies are very scarce, which motivated us to carry out relevant bioinformatics analysis.

In this study, we comprehensively analyzed the possible functions and mechanisms of APOBEC family members APOBEC1, APOBEC3A, APOBEC3G and APOBEC3H in the occurrence and development of PAAD by using public databases and a variety of bioinformatics analysis techniques. First, we analyzed the difference in APOBEC1/3A/3G/3H expression between pancreatic adenocarcinoma samples, matched adjacent samples and normal pancreatic tissues using The Cancer Genome Atlas (TCGA) and Genotype-Tissue Expression (GTEx) databases. Then, based on the R (version 3.6.3) and Kaplan‒Meier Plotter databases, we analyzed the relationship between their expression levels and clinicopathological features and overall survival of patients with PAAD. Finally, the potential mechanism of APOBEC1/3A/3G/3H involved in the occurrence and development of PAAD was explored by gene variation, immune infiltration, gene enrichment and protein-protein interaction (PPI) analysis. In summary, our research reveals that APOBEC1/3A/3G/3H plays a carcinogenic role in the occurrence and development of PAAD and is expected to become a new biomarker and therapeutic target for this type of malignant tumor.

## Methods

### Ethics statement

This study was approved by the academic Committee of the Cancer Hospital of Chinese Academy of Medical Sciences and strictly followed the principles of the Helsinki Declaration. All the data in this study were retrieved from online databases, and no human or animal experiments were involved.

### Expression analysis

In this study, R (version 3.6.3) was used to analyze the expression level of APOBEC1/3A/3G/3H in cancerous and paracancerous tissues in the TCGA database and normal pancreatic tissues in the GTEx database (https://xenabrowser.net/datapages/). The statistical significance of APOBEC1/3A/3G/3H expression was evaluated by the Wilcoxon test and *p* < 0.05 was considered statistically significant. Then, the correlation between the expression levels of APOBEC1/3A/3G/3H and clinical variables was analyzed by R (version 3.6.3). The statistical significance of APOBEC1/3A/3G/3H expression was evaluated by Fisher’s test and *p* < 0.05 was considered statistically significant. In addition, we searched the immunohistochemical staining results of APOBEC3A/3G/3H in PAAD and normal pancreatic tissues using HPA database (www.proteinatlas.org). HPA database offering the possibility to explore the tissue-elevated proteomes in tissues and organs and to analyze tissue profiles for specific protein classes. Comprehensive lists of proteins expressed at elevated levels in the different tissues have been compiled to provide a spatial context with localization of the proteins in the subcompartments of each tissue and organ down to the single-cell level.

### Survival analysis and ROC curve drawing

The Kaplan‒Meier Plotter database (http://www.kmplot.com/) is an online survival analysis tool capable of performing univariate and multivariate survival analysis using any custom-generated data which was used to analyze the correlation between the expression of APOBEC1/3A/3G/3H and OS and RFS in pancreatic adenocarcinoma and *p* < 0.05 was considered statistically significant (Lánczky and Győrffy, 2021). The “survival” R package (version 2.38) was utilized to calculate log-rank *p* values, and *p* values below 0.05 were considered statistically significant. The “pROC” R package (version 1.17.0.1) and “ggplot2″ R package (version 3.3.3) were used to analyze and draw ROC curves. The values of the area under the ROC curve (AUC) were between 0.5 and 1 (Nagy et al., 2021).

### Gene variation analysis

The cBioPortal database (http://www.cbioportal.org/) provides a Web resource for exploring, visualizing, and analyzing multidimensional cancer genomics data. The portal reduces molecular profiling data from cancer tissues and cell lines into readily understandable genetic, epigenetic, gene expression, and proteomic events (Gao et al., 2013). The cBioPortal database was used to analyze the gene variation of APOBEC1/3A/3G/3H in pancreatic adenocarcinoma, and the correlation between the variation and some clinicopathological features was further determined. The statistical significance of the difference was evaluated by the chi-squared test and *p* < 0.05 was considered statistically significant.

### Immune infiltration analysis

TISIDB database (http://cis.hku.hk/TISIDB/) integrated multiple types of data resources in oncoimmunology and we can cross-check a gene of interest about its role in tumor–immune interactions through literature mining and high-throughput data analysis, and generate testable hypotheses and high-quality figures for publication (Ru et al., 2019). The TISIDB database was used to analyze the correlation between the expression levels of APOBEC1/3A/3G/3H and the infiltration level of immune infiltrating cells and the expression level of immune molecules in pancreatic adenocarcinoma. The difference was evaluated by Spearman’s test and *p* < 0.05 was considered statistically significant. Then, the relationship between the expression levels of APOBEC1/3A/3G/3H and immune score and matrix score was analyzed by the SangerBox analysis tool (http://sangerbox.com/tool). The statistical significance of the difference was evaluated by Spearman’s test and *p* < 0.05 was considered statistically significant.

### Gene enrichment analysis

The LinkedOmics database (http://www.linkedomics.org/) contains multiomics data and clinical data for 32 cancer types and a total of 11158 patients from TCGA project. It is also the first multiomics database that integrates mass spectrometry based global proteomics data generated by the Clinical Proteomic Tumor Analysis Consortium on selected TCGA tumor samples (Vasaikar et al., 2018). The 400 genes most closely related to APOBEC1/3A/3G/3H coexpression were selected by using the LinkedOmics database, and the volcano map was drawn by R (version 3.6.3). The first 50 gene heatmaps that were positively correlated with APOBEC1/3A/3G/3H expression were drawn by R (version 3.6.3) and annotated by GeneCards (https://www.genecards.org/). Metascape (https://metascape.org) is a web-based portal designed to provide a comprehensive gene list annotation and analysis resource for experimental biologists. In terms of design features, Metascape combines functional enrichment, interactome analysis, gene annotation, and membership search to leverage over 40 independent knowledgebases within one integrated portal (Zhou et al., 2019). The Metascape database visualization of APOBEC1/3A/3G/3H and their coexpressed genes Biological Process (BP), Cellular Components (CC), Molecular Function (MF) and Kyoto Encyclopedia of Genes and Genomes (KEGG) were used. Wayne diagrams of 8 genes coexpressed with APOBEC3A, APOBEC3G and APOBEC3H were drawn by R (version 3.6.3), and the eight genes were analyzed by GO and KEGG enrichment analysis. TIMER database (http://timer.cistrome.org/) provides 6 major analytic modules that allow users to interactively explore the associations between immune infiltrates and a wide-spectrum of factors, including gene expression, clinical outcomes, somatic mutations, and somatic copy number alterations (Mermel et al., 2011; Li et al., 2017). The correlation between APOBEC1/3A/3G/3H expression levels was analyzed by the TIMER database. The statistical significance of the difference was evaluated by Spearman’s test and *p* < 0.05 was considered statistically significant. In addition, the pathway enrichment of APOBEC1/3A/3G/3H was completed by using the GSCALite database (http://bioinfo.life.hust.edu.cn/web/GSCALite/). GSCALite database is a user-friendly web server for dynamic analysis and visualization of gene set in cancer and drug sensitivity correlation, which will be of broad utilities to cancer researchers (Liu et al., 2018).

### Construction of the functional PPI network

Functional links between proteins can often be inferred from genomic associations between the genes that encode them: groups of genes that are required for the same function tend to show similar species coverage, are often located in close proximity on the genome (in prokaryotes), and tend to be involved in gene-fusion events. The STRING database (https://string-db.org/) is a precomputed global resource for the exploration and analysis of these associations (Von Mering et al., 2003). The genes with the strongest interaction with APOBEC1/3A/3G/3H proteins were obtained by using the STRING database, and the functional protein interaction network of APOBEC1/3A/3G/3H was established. Additionally, the interaction intensity of different genes in the PPI network was scored by Cytoscape (version 3.9.1) software (Shannon et al., 2003; Doncheva et al., 2019).

### Drug sensitivity analysis

The GSCALite database was used to analyze the correlation between the expression level of APOBEC3G and the sensitivity of many kinds of chemotherapy or targeted therapies. The difference was evaluated by Spearman’s test and *p* < 0.05 was considered statistically significant.

## Results

### Aberrant expression of APOBEC1, APOBEC3A, APOBEC3G and APOBEC3H across cancers

**FIGURE 1:**
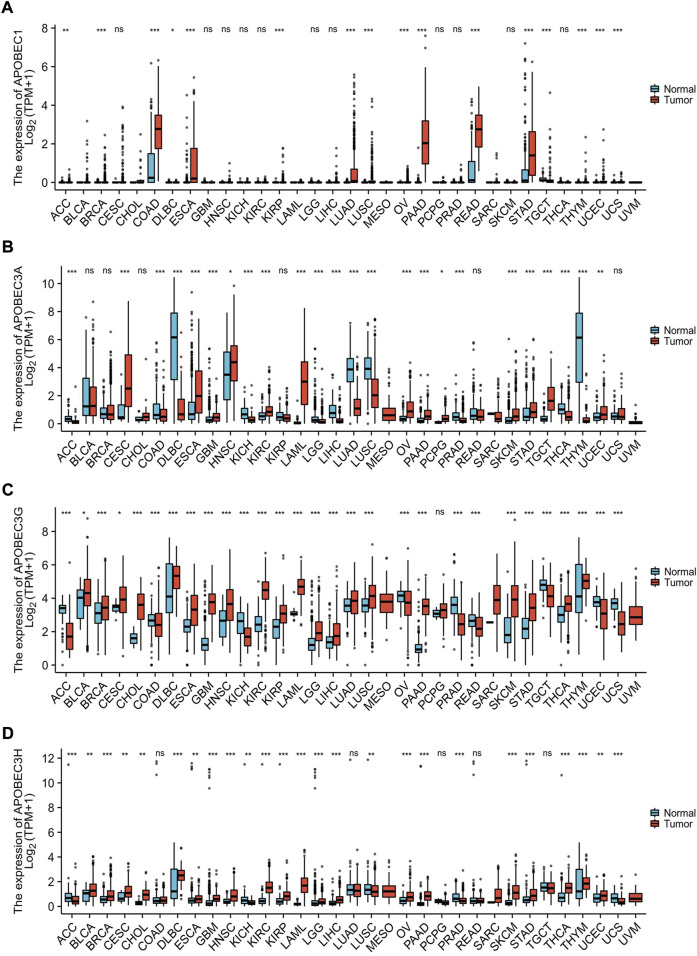
Expression analysis of the APOBEC family members APOBEC1, APOBEC3A, APOBEC3G and APOBEC3H across cancers. **(A)** The expression level of APOBEC1; **(B)** The expression level of APOBEC3A; **(C)** The expression level of APOBEC3G; **(D)** The expression level of APOBEC3H. ns, *p* ≥ 0.05; *, *p* < 0.05; **, *p* < 0.01; ***, *p* < 0.001.

Based on the data from the TCGA database and GTEx database, we analyzed the transcription levels of APOBEC1/3A/APOBEC3G/3H in many kinds of cancer tissues, adjacent tissues and normal tissues. The results showed that the transcription levels of APOBEC1/3A/3G/3H in 28 kinds of cancer tissues, including PAAD, esophageal carcinoma (ESCA) and stomach adenocarcinoma (STAD), was higher than that in paracancerous tissues and normal tissues but lower (Figures 1A–D) in 13 other kinds of cancer tissues, including lymphoid neoplasm diffuse large B-cell lymphoma (DLBC), kidney chromophobe (KICH) and lung squamous cell carcinoma (LUSC). The above findings suggest that there are differences in the expression of APOBEC1/3A/3G/3H across cancers and may play different roles in different cancers. In addition, we searched the immunohistochemical staining results of APOBEC3A/3G/3H in PAAD (Supplementary Figures S2A,C,E) and normal pancreatic tissues (Supplementary Figures S2B,D,F) using the HPA database.

### Relationship between high expression levels of APOBEC1, APOBEC3A, APOBEC3G and APOBEC3H and clinicopathological features and prognosis in patients with PAAD

**FIGURE 2:**
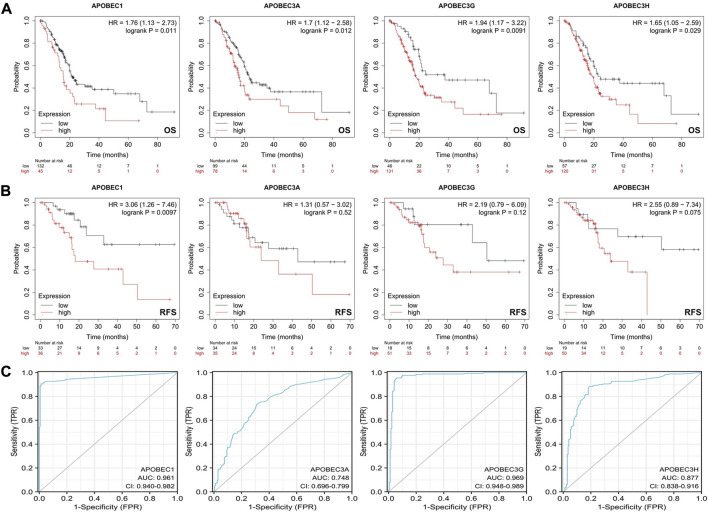
Survival analysis of the APOBEC family members APOBEC1, APOBEC3A, APOBEC3G and APOBEC3H in PAAD. **(A)** The increased expression levels of APOBEC1/3A/3G/3H were significantly correlated with shorter OS; **(B)** The increased expression level of APOBEC1 was significantly correlated with shorter RFS; **(C)** APOBEC1/3A/3G/3H showed high accuracy in predicting normal and neoplastic outcomes.

First, we analyzed the relationship between the expression levels of APOBEC1/3A/3G/3H and the clinicopathological characteristics of PAAD patients based on the data from the PAAD project in the TCGA database and R (version 3.6.3). As shown in Table 1, high expression of APOBEC1 was significantly correlated with higher pathologic stage (*p* < 0.020), and high expression of APOBEC3H was significantly correlated with higher N stage (*p* < 0.003). Next, we analyzed the correlation between the expression level of APOBEC1/3A/3G/3H and the prognosis of patients with PAAD through the Kaplan‒Meier Plotter database. The results showed that the high expression of APOBEC1/3A/3G/3H was significantly correlated with shorter overall survival (OS) (Figure 2A), and the high expression of APOBEC1 was also significantly correlated with shorter recurrence-free survival (RFS) (*p* < 0.05) (Figure 2B). Finally, we drew ROC curves based on data from the TCGA and GTEx databases to distinguish normal and pancreatic adenocarcinoma tissues, and APOBEC1/3A/3G/3H showed high accuracy in predicting normal and tumor outcomes. The ROC curve Figure 2C shows that APOBEC1 AUC is 0.961, APOBEC3A AUC is 0.748, APOBEC3A AUC is 0.696–0.799, AUC is 0.969, AUC is 0.87, and AUC is 0.838–0.916. In summary, these results show that APOBEC1/3A/3G/3H is generally upregulated in PAAD, and the high expression levels of APOBEC1/3A/3G/3H are related to the poor clinicopathological features and prognosis of PAAD patients. These results also indicate the potential role of APOBEC1/3A/3G/3H in the occurrence and progression of PAAD, suggesting that APOBEC1/3A/3G/3H can be used as a prognostic marker in PAAD patients.

**TABLE 1:** Relationship between the APOBEC family members APOBEC1, APOBEC3A, APOBEC3G and APOBEC3H expression and clinicopathological characteristics of PAAD patients.

Characteristic	APOBEC1 expression		*p*-value	APOBEC3A expression		*p*-value	APOBEC3G expression		*p*-value	APOBEC3H expression		*p*-value
	High n (%)	Low n (%)		High n (%)	Low n (%)		High n (%)	Low n (%)		High n (%)	Low n (%)	
Histologic grade												
G1	14 (7.95)	17 (9.66)	0.573	14 (7.95)	17 (9.66)	0.919	15 (8.52)	16 (9.09)	0.821	13 (7.39)	18 (10.23)	0.508
G2	50 (28.41)	45 (25.57)		49 (27.84)	46 (26.14)		45 (25.57)	50 (28.41)		46 (26.14)	49 (27.84)	
G3	24 (13.64)	24 (13.64)		24 (13.64)	24 (13.64)		27 (15.34)	21 (11.93)		28 (15.91)	20 (11.36)	
G4	0 (0.00)	2 (1.14)		1 (0.57)	1 (0.57)		1 (0.57)	1 (0.57)		1 (0.57)	1 (0.57)	
Pathologic stage												
Stage I	5 (2.86)	16 (9.14)	0.020	8 (4.57)	13 (7.43)	0.678	10 (5.71)	11 (6.29)	0.499	6 (3.43)	15 (8.57)	0.120
Stage II	78 (44.57)	68 (38.86)		75 (42.86)	71 (40.57)		77 (44.00)	69 (39.43)		79 (45.14)	67 (38.29)	
Stage III	1 (0.57)	2 (1.14)		2 (1.14)	1 (0.57)		1 (0.57)	2 (1.14)		2 (1.14)	1 (0.57)	
Stage IV	4 (2.29)	1 (0.57)		2 (1.14)	3 (1.71)		1 (0.57)	4 (2.29)		2 (1.14)	3 (1.71)	
T stage												
T1	3 (1.70)	4 (2.27)	0.465	2 (1.14)	5 (2.84)	0.479	2 (1.14)	5 (2.84)	0.540	1 (0.57)	6 (3.41)	0.135
T2	9 (5.11)	15 (8.52)		10 (5.68)	14 (7.95)		14 (7.95)	10 (5.68)		10 (5.68)	14 (7.95)	
T3	76 (43.18)	66 (37.50)		74 (42.05)	68 (38.64)		72 (40.91)	70 (39.77)		76 (43.18)	66 (37.50)	
T4	1 (0.57)	2 (1.14)		2 (1.14)	1 (0.57)		1 (0.57)	2 (1.14)		2 (1.14)	1 (0.57)	
N stage												
N0	23 (13.29)	27 (15.61)	0.581	21 (12.14)	29 (16.76)	0.304	22 (12.72)	28 (16.18)	0.325	16 (9.25)	34 (19.65)	0.003
N1	64 (37.00)	59 (34.10)		64 (36.99)	59 (34.10)		66 (38.15)	57 (32.95)		72 (41.62)	51 (29.48)	
M stage												
M0	37 (44.05)	42 (50.00)	0.197	45 (53.57)	34 (40.48)	0.650	45 (53.57)	34 (40.48)	0.171	46 (54.76)	33 (39.29)	0.647
M1	4 (47.62)	1 (1.19)		2 (2.38)	3 (3.57)		1 (1.19)	4 (4.76)		2 (2.38)	3 (3.57)	

Notes: Bold numbers indicate *p* < 0.05.

### Relationship between gene variation of APOBEC1, APOBEC3A, APOBEC3G and APOBEC3H and clinicopathological features of PAAD patients

**FIGURE 3:**
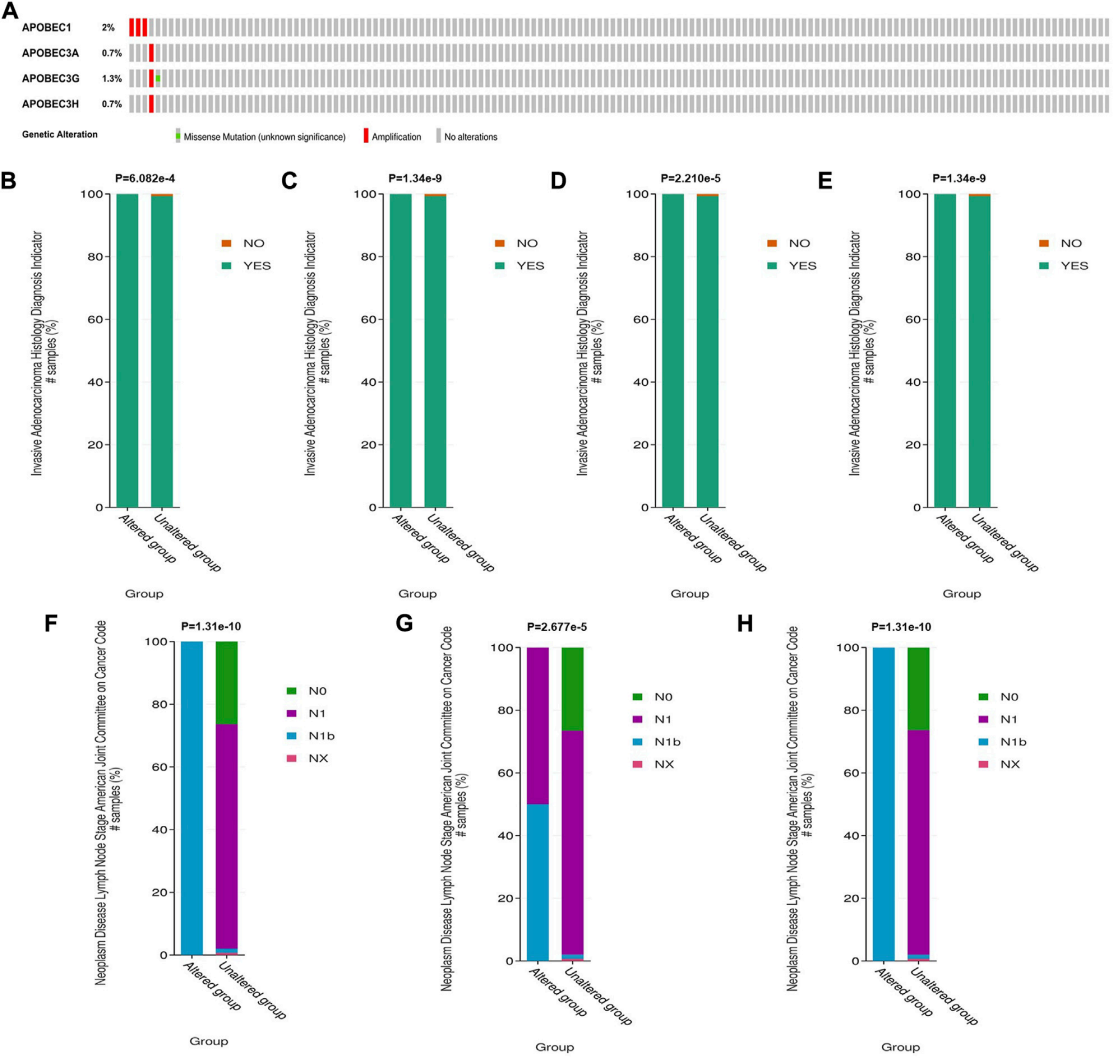
Genetic variation analysis of the APOBEC family members APOBEC1, APOBEC3A, APOBEC3G and APOBEC3H in PAAD. **(A)** Gene variation characteristics of the APOBEC1/3A/3G/3H in PAAD; **(B–E)** The amplification variants of APOBEC1/3A/3G/3H were significantly correlated with PAAD invasion of surrounding tissues; **(F–H)** The amplification variants of APOBEC3A/3G/3H were significantly correlated with Higher N stage of PAAD.

To further explore the mechanism of the differential expression of APOBEC1/3A/3G/3H in pancreatic adenocarcinoma, we used the cBioPortal online tool to analyze the gene variation of APOBEC1/3A/3G/3H. APOBEC1/3A/3G/3H had genetic variation in 5 samples (3%) from patients with pancreatic adenocarcinoma, of which the gene with the highest frequency of mutation is APOBEC1 (2%), and the main type of variation is amplification (Figure 3A). Based on this, we analyzed the clinicopathological features of patients with APOBEC1/3A/3G/3H gene mutation and nonmutation PAAD. The results showed that there was a significant correlation between the amplification variation of APOBEC1/3A/3G/3H and the invasion of surrounding tissues of pancreatic adenocarcinoma (Figures 3B–E). There was also a significant correlation between the amplification variations of APOBEC3A/3G/3H and the higher N stage of PAAD patients (Figures 3F–H). The above results suggest that APOBEC1/3A/3G/3H will be amplified and mutated in PAAD tissues, which will lead to an increase in APOBEC1/3A/3G/3H expression and poor clinicopathological features of PAAD patients, which may be another important factor leading to worse prognosis in PAAD patients.

### Immune landscape of APOBEC1, APOBEC3A, APOBEC3G and APOBEC3H in patients with PAAD

**FIGURE 4:**
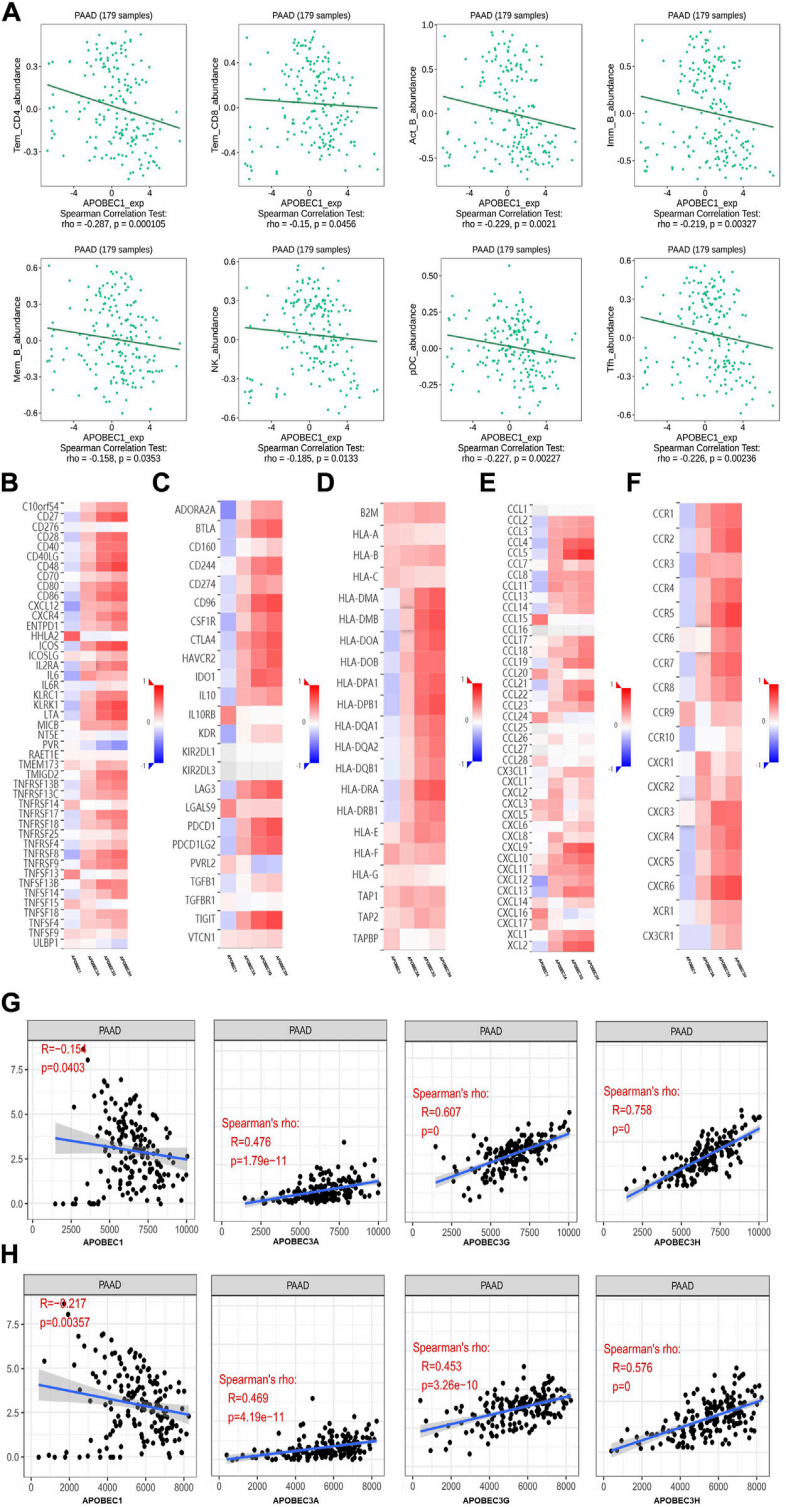
The immune landscape of the APOBEC family members APOBEC1, APOBEC3A, APOBEC3G and APOBEC3H in PAAD patients. **(A)** APOBEC1 was negatively correlated with the infiltration levels of various immunocompetent tumor infiltrating cells including Tem_CD4, Tem_CD8 and NK; **(B–F)** APOBEC1 was negatively correlated with the expression levels of most immune promoters and APOBEC3A/3G/3H were positively correlated with most immunomodulators (immune promoters, MHC molecules, chemokines, and chemokine receptors). **(G)** APOBEC1 was negatively correlated with the immune score and APOBEC3A/3G/3H were positively correlated with the immune score; **(H)** APOBEC1 was negatively correlated with the matrix score and APOBEC3A/3G/3H were positively correlated with the matrix score.

First, we used the data from the PAAD project in the TISIDB database to explore the relationship between APOBEC1/3A/3G/3H expression and the level of tumor infiltrating cells and multiple immunomodulators based on various immunological markers in PAAD. Our results show that the roles of APOBEC1/3A/3G/3H in tumor immune regulation are not consistent. First, APOBEC1 was negatively correlated with the infiltration level of many kinds of immunoreactive tumor-infiltrating cells, including Tem_CD4, Tem_CD8 and NK cells (Figure 4A), and negatively correlated with the expression level of most immune promoters (Figure 4B). Therefore, the high expression of APOBEC1 may inhibit the immune response to PAAD, which may be an important factor leading to immune escape of PAAD cells. In contrast, APOBEC3A/3G/3H were positively correlated with the infiltration level of many kinds of immunoreactive tumor-infiltrating cells, including Tem_CD4, Tem_CD8 and NK cells (Supplementary Figure S1), and positively correlated with the expression level of most immunomodulators (immunoenhancers, MHC molecules, chemokines and chemokine receptors) in PAAD (Figures 4A,C–F), indicating that APOBEC3A/3G/3H may play immune-promoting roles in the PAAD tumor microenvironment.

Then, based on the data from the PAAD project in the TCGA database, we used the SangerBox analysis tool to further explore the relationship between APOBEC1/3A/3G/3H expression levels and immune scores and matrix scores. The results showed that APOBEC1 was negatively correlated with the immune score and matrix score of PAAD, while APOBEC3A/3G/3H showed the opposite correlation (Figures 4G,H).

These results suggest that the role of the APOBEC family in the immune regulation of the PAAD tumor microenvironment is complex and diverse. Special attention should be given to the immune escape of PAAD cells caused by high expression of APOBEC1, which may be a potential target for the treatment of PAAD.

### Enrichment analysis of APOBEC1, APOBEC3A, APOBEC3G, APOBEC3H and the 400 coexpressed genes

**FIGURE 5:**
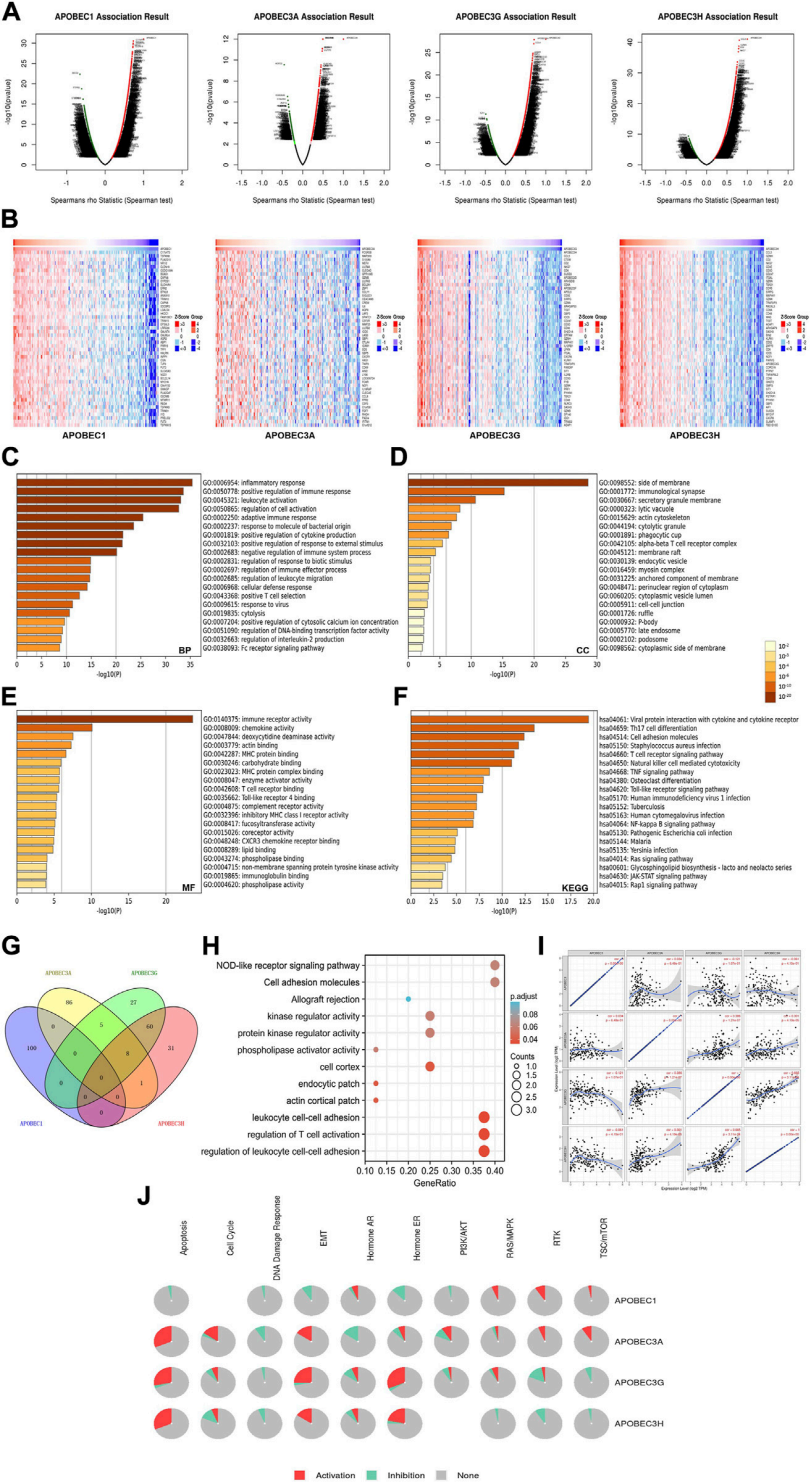
Gene enrichment analysis of the APOBEC family members APOBEC1, APOBEC3A, APOBEC3G and APOBEC3H in PAAD. **(A)** The volcano plot of APOBEC1/3A/3G/3H and their 400 coexpressed genes; **(B)** The top 50 genes positively correlated with APOBEC1/3A/3G/3H expression; **(C–E)** The GO enrichment of the BP terms, CC terms and MF terms of APOBEC1/3A/3G/3H and their 400 coexpressed genes; **(F)** The KEGG enrichment of APOBEC1/3A/3G/3H and their 400 coexpressed genes; **(G)** The venn diagram of APOBEC1/3A/3G/3H and their 400 coexpressed genes; **(H)** GO and KEGG enrichment analysis of 8 genes co-expressed with APOBEC3A, APOBEC3G and APOBEC3H; **(I)** APOBEC1 was negatively correlated with the expression levels of APOBEC3G and APOBEC3H; **(J)** APOBEC1/3A/3G/3H played an activating role in a variety of oncogenic pathways.

To further explore the mechanisms of APOBEC1/3A/3G/3H in the occurrence and development of PAAD, we first obtained 400 coexpressed genes (Supplementary Material S1) of APOBEC1/3A/3G/3H in pancreatic adenocarcinoma from the LinkedOmics database and generated the volcano map (Figure 5A). We displayed the first 50 genes that were positively related to the APOBEC1/3A/3G/3H table in the heatmaps (Figure 5B) and then annotated them with GeneCards. The results showed that many oncogenes, including MCU, MAP3K8, GZMK and TNFAIP8L2, were positively correlated with the expression of APOBEC1/3A/3G/3H.

Then, we carried out GO and KEGG enrichment analyses of APOBEC1/3A/3G/3H and their 400 coexpressed genes, which were based on the data from the PAAD project in the Metascape database. GO analysis showed that APOBEC1/3A/3G/3H and their 400 coexpressed genes mainly acted on BP terms (Figure 5C), such as “inflammatory response”, “positive regulation of immune response” and “leukocyte activation”; CC terms, such as “side of membrane”, “immunological synapse” and “secretory granule membrane” (Figure 5D); and MF terms, such as “immune receptor activity”, “chemokine activity” and “deoxycytidine deaminase activity” (Figure 5E). KEGG analysis showed that APOBEC1/3A/3G/3H and their 400 coexpressed genes may play important roles in many pathways related to immune activation, such as the “T-cell receptor signaling pathway”, “TNF signaling pathway”, and the “Tolllike receptor signaling pathway” (Figure 5F).

Then, we obtained eight genes (ICOS, GBP5, TIGIT, GMFG, CCL5, CYTIP, SP140 and PRF1) coexpressed with APOBEC3A, APOBEC3G and APOBEC3H by drawing a Wayne diagram (Figure 5G) and then carried out GO and KEGG enrichment analyses of these eight genes (Figure 5H). The results showed that the target genes mainly acted on “regulation of leukocyte cell‒cell adhesion”, “regulation of T-cell activation”, “leukocyte cell‒cell adhesion” and “NOD-like receptor signaling pathway”. In addition, we also found that the expression level of APOBEC1 was negatively correlated with APOBEC3G and APOBEC3H (cor = -0.121/cor = -0.061), while the coexpression correlation between APOBEC3G and APOBEC3H was higher (cor = 0.665) (Figure 5J).

Finally, we further analyzed the mechanisms of APOBEC1/3A/3G/3H in many carcinogenic pathways by using PAAD project data from the GSCA database. As shown in Figure 5J, APOBEC3A/3G/3H play an activating role in the “EMT signaling pathway”, and APOBEC3A is also an activating factor of the “RTK signaling pathway” and “TSC/mTOR signaling pathway”. APOBEC1 plays an active role in the “RAS/MAPK signaling pathway”, “RTK signaling pathway” and “TSC/mTOR signaling pathway".

In summary, these results further indicate that APOBEC1/3A/3G/3H play important roles in the immune regulation of the tumor microenvironment in PAAD. In addition, the coexpression of APOBEC1/3A/3G/3H and a variety of tumor-promoting genes and their activation of a variety of tumor-promoting pathways may be a potential mechanism for promoting the occurrence and development of PAAD.

### Construction and analysis of the PPI network related to APOBEC1, APOBEC3A, APOBEC3G and APOBEC3H

**FIGURE 6:**
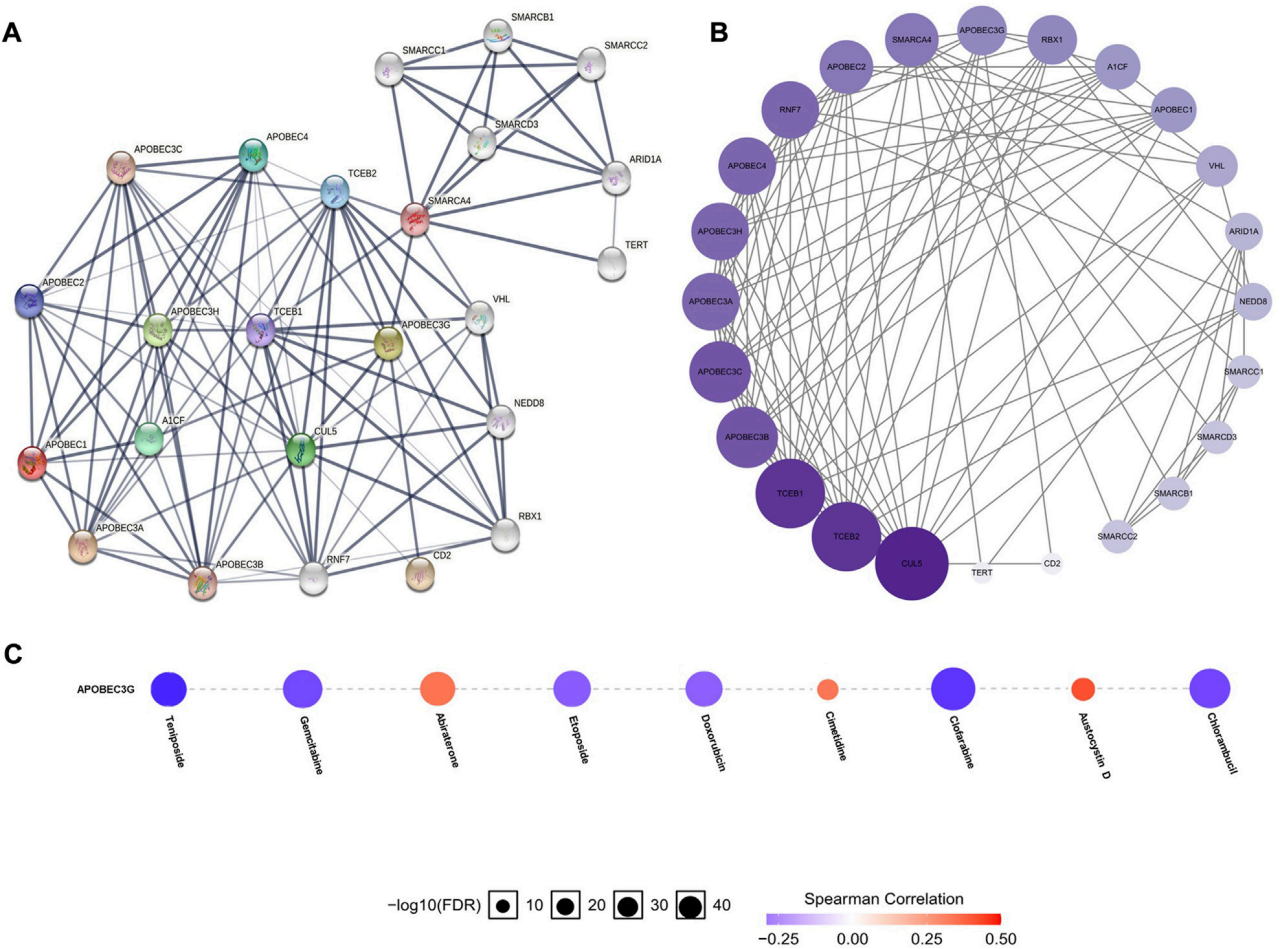
PPI network construction of the APOBEC family members APOBEC1, APOBEC3A, APOBEC3G and APOBEC3H and drug sensitivity analysis of APOBEC3G in PAAD. **(A,B)** SMARCA4 and CUL5 played an important role in PPI network which was closely related to APOBEC1/3A/3G/3H; **(C)** The expression level of APOBEC3G was positively correlated with the sensitivity of various targeted or chemotherapeutic drugs, including gemcitabine and doxorubicin.

To construct and analyze the PPI network of APOBEC1/3A/3G/3H in patients with pancreatic adenocarcinoma, we used the STRING database to identify 23 genes (Figure 6A) (Supplementary Material S2) with the strongest PPI with APOBEC1/3A/3G/3H and then drew the related PPI network with Cytoscape software, in which the larger the circle and darker the color, the greater the number of PPIs associated with the gene. The results showed that CUL5 had a higher score (Figure 6B) (Supplementary Material S3) in the APOBEC1/3A/3G/3H PPI network, which indicates that CUL5 plays an important role in this PPI network, which is closely related to APOBEC1/3A/3G/3H. Another interesting finding is that SMARCA4 may be the key gene (Figure 6A) that induces the occurrence of PPI between the SWI/SNF family and APOBEC family.

### The expression level of APOBEC3G affects the sensitivity of multiple drugs

Finally, we used the GSCALite online database to analyze the expression level of APOBEC3G and the sensitivity of a variety of chemotherapy and targeted drugs. As shown in Figure 6C, the expression level of APOBEC3G was positively correlated with the sensitivity to many targeted or chemotherapeutic drugs, including gemcitabine and doxorubicin. Therefore, gemcitabine and doxorubicin may have a better therapeutic effect in PAAD patients with high APOBEC3G expression.

## Discussion

APOBEC proteins are a “double-edged sword”; they act as antiviral factors, and their overexpression or sustained expression may induce carcinogenicity and lead to a higher mutation load in a variety of human tumors, including bladder cancer, lung cancer, head and neck cancer and breast cancer (Iizuka et al., 2017). Although some studies have revealed the carcinogenic role of APOBEC family members APOBEC1, APOBEC3A, APOBEC3G and APOBEC3H in esophageal cancer, breast cancer, liver cancer and head and neck squamous cell carcinoma (Yamanaka et al., 1995; Saraconi et al., 2014; Kim et al., 2020; Liu Q et al., 2020), their function in PAAD is still unclear; additionally, there are very few related studies and specific bioinformatics analysis has not been carried out. This study is the first bioinformatics analysis of the function of APOBEC1/3A/3G/3H in PAAD. It reveals the function and possible mechanisms of APOBEC1/3A/3G/3H in the occurrence and development of PAAD from the aspects of gene expression, gene variation, immune infiltration, gene enrichment, protein interaction and drug sensitivity.

APOBEC1 can deaminate single-stranded DNA or RNA, and its deamination activity is related to cancer (Wolfe et al., 2020). In addition, overexpression of APOBEC1 leads to the editing of additional cytidine sites in the substrate, which is considered to be the main cause of abnormal mutation in cancer cells (Fujino et al., 1998). It has been reported that high expression of APOBEC1 is associated with poor prognosis of adrenocortical carcinoma, endometrial carcinoma, mesothelioma and thyroid carcinoma (Niavarani et al., 2018). In this study, we found that APOBEC1 was also highly expressed in PAAD tissues and was significantly associated with higher PAAD tumor grade and shorter OS and RFS. Therefore, APOBEC1 may lead to a worse prognosis of PAAD patients through the same mechanism. In addition, APOBEC1 can not only enhance genomic variation by introducing intraframe genomic insertions and deletions into normal pluripotent cells, leading to cancer (Niavarani et al., 2018), but our study also found that the APOBEC1 gene itself can have amplification mutations within the PAAD tissue; this will lead to increased expression of APOBEC1, resulting in pancreatic adenocarcinoma being more invasive and may be another important factor leading to poor prognosis in PAAD patients. Another interesting finding is that APOBEC1 plays an active role in the “RAS/MAPK signaling pathway”, “RTK signaling pathway” and “TSC/mTOR signaling pathway”. The “RAS/MAPK signaling pathway” and “RTK signaling pathway” can promote the growth and proliferation of tumor cells (Ledda and Paratcha, 2007; Rezatabar et al., 2019), while the “TSC/mTOR signaling pathway” can promote tumor angiogenesis (Jham et al., 2011), which may be another mechanism by which APOBEC1 plays a carcinogenic role.

APOBEC3A, APOBEC3G and APOBEC3H have similar functions; their catalytic activity is necessary for DNA damage activation (Landry et al., 2011) and has strong antiviral activity (Wang et al., 2008). However, some studies have found that this antiviral ability may be at the expense of an increased risk of host genome changes, and uncontrolled genomic deamination is potentially harmful and may be the cause of genomic instability and cancer (Landry et al., 2011). Related studies have shown that the high expression of APOBEC3A/3G/3H is related to the occurrence and development of colon cancer, multiple myeloma and head and neck squamous cell carcinoma (Wang et al., 2008; Ding et al., 2011; Liu J et al., 2020). Our study found that APOBEC3A/3G/3H are also highly expressed in PAAD tissues, and the APOBEC3A/3G/3H gene itself can be amplified and mutated, which leads to an increase in the expression levels of APOBEC3A/3G/3H, resulting in uncontrolled deamination of the cellular genome. This potential mechanism may be an important factor leading to more aggressive PAAD and worse prognosis of patients. The “EMT signaling pathway” is a classical cancer-promoting pathway that leads to the formation of secondary metastatic lesions by activating the motor and invasive abilities of tumor cells. It plays a cancer-promoting role in many kinds of tumors, including pancreatic adenocarcinoma, prostate cancer and breast cancer (Kong et al., 2021; Nowak and Bednarek, 2021; Zhao et al., 2021; Peng et al., 2022). The activation of APOBEC3A/3G/3H in the “EMT signaling pathway” may be another mechanism to promote the occurrence and development of PAAD.

Although cancer immunotherapy has been shown to improve the survival rate of many kinds of cancer patients, the remission rate of PAAD patients is still very low (Ribas and Wolchok, 2018). Therefore, it is important to find new immunotherapy targets and develop new immunotherapy strategies. We analyzed the immune characteristics of APOBEC1/3A/3G/3H in PAAD. The results showed that APOBEC1 was negatively correlated with the infiltration level of many kinds of immunoreactive tumor infiltrating cells, the expression level of most immune promoters, immune score and matrix score, indicating that the high expression of APOBEC1 may inhibit the immune response to PAAD, while the immunomodulatory effects of APOBEC3A/3G/3H are the opposite. Previous studies have found that APOBEC3A/3G/3H play important immune-promoting roles in the innate immune system and have antiviral activity against a variety of retroviruses (Wang et al., 2008; Landry et al., 2011; Garg et al., 2015), which is consistent with our GO and KEGG analysis results. However, the antiviral activity of APOBEC3A/3G/3H is achieved by targeted deamination of cytidine residues of single-stranded DNA produced during viral genomic RNA reverse transcription. Overexpression of APOBEC3A/3G/3H may lead to DNA fragmentation and increase genomic instability, which may lead to cancer risk (Wang et al., 2008). Therefore, the immunosuppressive effect of APOBEC1 in the PAAD tumor microenvironment and the genomic instability caused by the high expression of APOBEC3A/3G/3H in PAAD tissues may be important factors in the occurrence and development of PAAD. These results suggest that APOBEC1/3A/3G/3H can be used as potential targets for PAAD immunotherapy and as a molecular index for predicting the efficacy of immunotherapy.

Another interesting finding is the strong PPI between CUL5 and SMARCA4 and APOBEC1/3A/3G/3H. The CUL5 protein is involved in the formation of the E3-specific ligase complex and is responsible for ubiquitin protein transport to its target substrate for ubiquitin-dependent degradation (Li et al., 2021). The traditional view is that CUL5 is a potential tumor suppressor that can inhibit the proliferation, migration and invasion of renal cell carcinoma, endometrial carcinoma, prostate cancer, gastric cancer and lung cancer cells (Zhu et al., 2019; Wang et al., 2020) and maintains genomic stability (Yu et al., 2003). Recent studies have found that the complex composed of CUL5 can induce ubiquitination and degradation of APOBEC3G (Yu et al., 2003), which may be the mechanism for maintaining genomic stability. Our analysis shows that there is also a strong PPI between CUL5 and APOBEC1/3A/3H, suggesting that CUL5 may also avoid excessive damage to APOBEC1/3A/3H by inducing ubiquitin and degradation of DNA; this needs to be verified by further research. SMARCA4, a member of the SWI/SNF protein family, has helicase and ATP enzyme activities and can regulate the transcription of some genes by changing the structure of the surrounding chromatin (Jubierre et al., 2016). Some studies have found that SMARCA4 is highly expressed in pancreatic adenocarcinoma and participates in many processes, such as cancer cell growth and proliferation (Jubierre et al., 2016). The strong PPI between SMARCA4 and APOBEC1/3A/3G/3H may be the synergistic effect that promotes the occurrence and development of PAAD, which also needs be verified through further research.

In terms of clinical transformation, our study found that the expression level of APOBEC3G was positively correlated with the sensitivity to a variety of targeted or chemotherapeutic drugs, including gemcitabine and doxorubicin. Therefore, gemcitabine or doxorubicin may have a better therapeutic effect in PAAD patients with high APOBEC3G expression.

This study is the first bioinformatics analysis of the function of APOBEC family members APOBEC1, APOBEC3A, APOBEC3G and APOBEC3H in PAAD. We found that there are differences in the expression of APOBEC1/3A/3G/3H across cancers, and may play different roles in different cancers; however, the high expression and amplification variation of APOBEC1/3A/3G/3H are significantly related to worse clinicopathological features and prognosis of PAAD patients. In addition, APOBEC1/3A/3G/3H may promote the occurrence and development of PAAD by activating a variety of carcinogenic pathways and regulating PAAD tumor immunity. Another important finding is the possible synergy between SMARCA4 and APOBEC1/3A/3G/3H in promoting the occurrence and development of PAAD. In terms of clinical transformation, our study found that gemcitabine or doxorubicin may have a better therapeutic effect in PAAD patients with high expression of APOBEC3G. However, this study has some limitations. For example, the number of databases included in this study is somewhat insufficient. In addition, this study is only a bioinformatics analysis of the function and mechanism of APOBEC1/3A/3G/3H in the occurrence and development of PAAD. Future experimental studies can further confirm the tumor-promoting role of APOBEC1/3A/3G/3H in PAAD.

## Conclusion

In short, our results show that the APOBEC family members APOBEC1, APOBEC3A, APOBEC3G and APOBEC3H may play a carcinogenic role in the occurrence and development of PAAD and are expected to become new biomarkers and therapeutic targets of PAAD. However, further studies are needed to verify our findings and to promote the clinical application of APOBEC1, APOBEC3A, APOBEC3G and APOBEC3H in PAAD.

## Data Availability

The original contributions presented in the study are included in the article/Supplementary Material, further inquiries can be directed to the corresponding author.

## Abbreviations

APOBEC, Apolipoprotein B mRNA-editing catalytic polypeptide; BP, Biological process; CC, Cellular components; DLBC, Lymphoid neoplasm diffuse large B-cell lymphoma; ESCA, Esophageal carcinoma; GTEx, Genotype-Tissue Expression; KEGG, Kyoto Encyclopedia of Genes and Genomes; KICH, Kidney chromophobe; LUSC, Lung squamous cell carcinoma; MF, Molecular function; OS, Overall survival; PAAD, Pancreatic adenocarcinoma; PPI, Protein-protein interaction; RFS: Recurrence-free survival; STAD, Stomach adenocarcinoma; TCGA, The Cancer Genome Atlas; ZDD, Zinc-dependent cytidine deaminase domain.
