# Supplementary figures and images for: Prognostic value, immune signature and molecular mechanisms of the APOBEC family members APOBEC1, APOBEC3A, APOBEC3G and APOBEC3H in pancreatic adenocarcinoma
Source: Front Mol Biosci. 2022 Oct 20;9:1036287. doi: 10.3389/fmolb.2022.1036287 (PMC9631948; doi:10.3389/fmolb.2022.1036287)

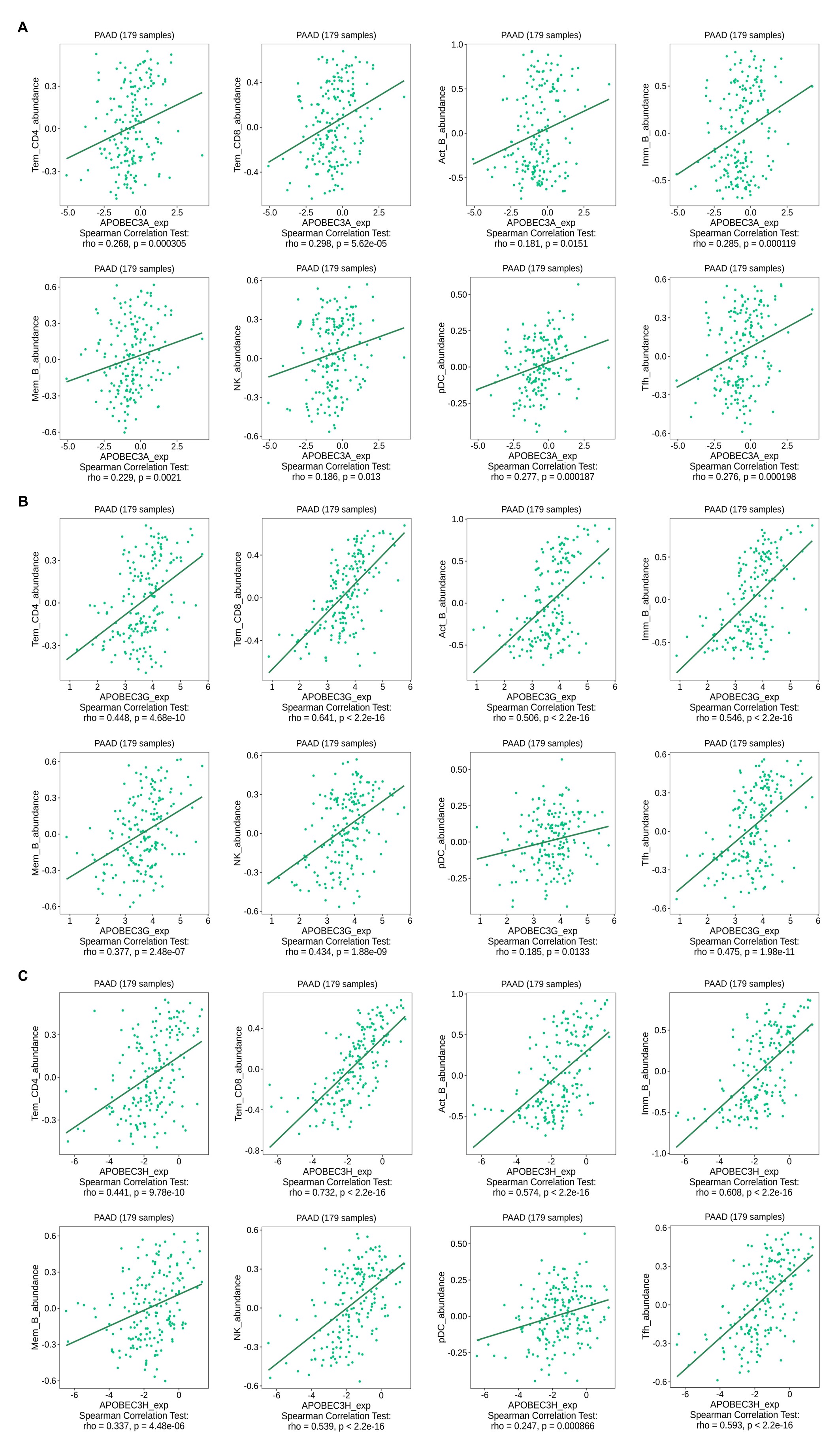

Supplement: Supplementary file 3 [file Image1.JPEG]

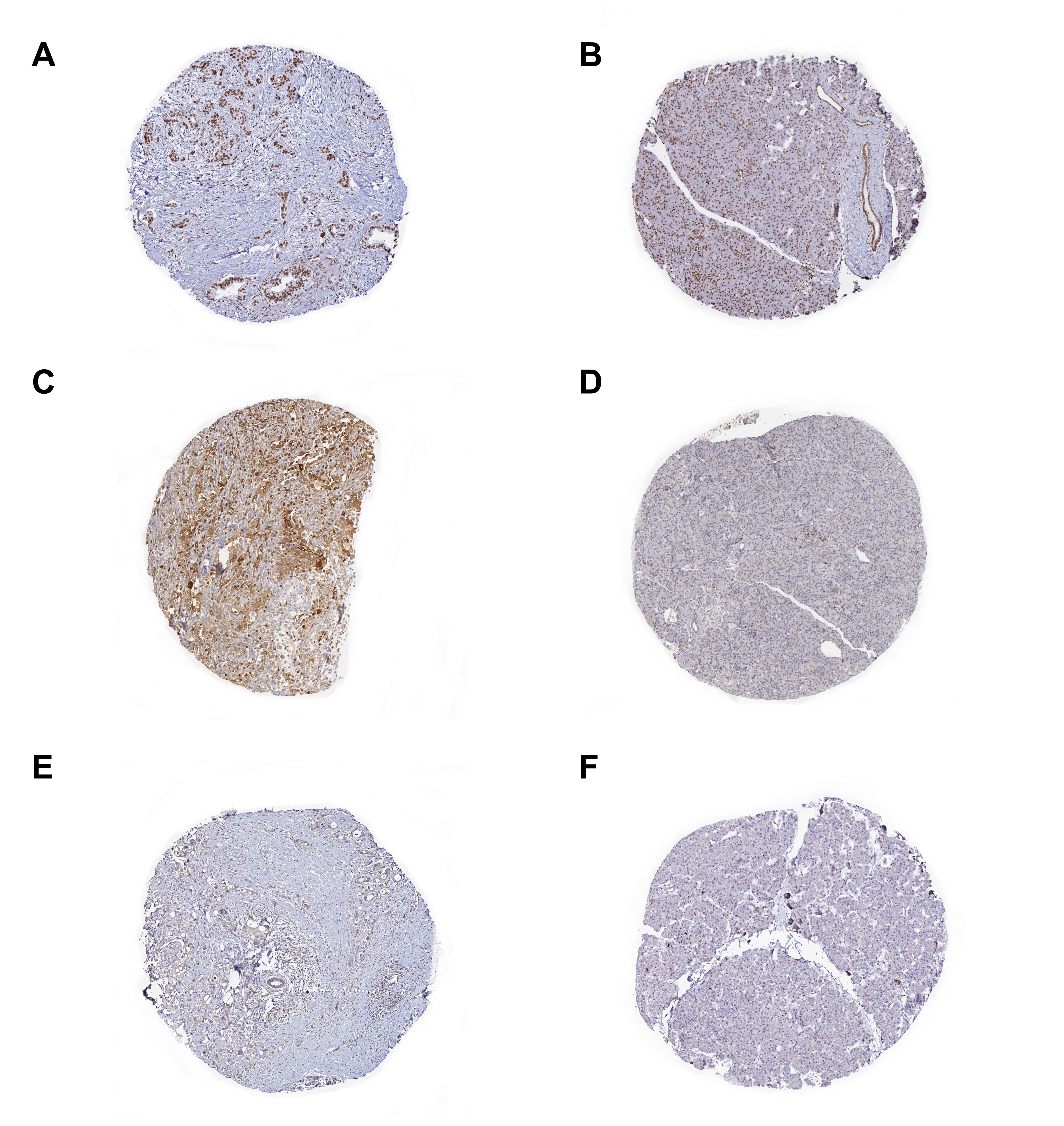

Supplement: Supplementary file 4 [file Image2.JPEG]
